# Blood brain barrier disruption and glutamatergic excitotoxicity in post-acute sequelae of SARS COV-2 infection cognitive impairment: potential biomarkers and a window into pathogenesis

**DOI:** 10.3389/fneur.2024.1350848

**Published:** 2024-05-02

**Authors:** Joga Chaganti, Govinda Poudel, Lucette Adeline Cysique, Gregory J. Dore, Anthony Kelleher, Gael Matthews, David Darley, Anthony Byrne, David Jakabek, Xin Zhang, Marrissa Lewis, Nikhil Jha, Bruce James Brew

**Affiliations:** ^1^Thomas Jefferson University, Philadelphia, PA, United States; ^2^Mary MacKillop Institute for Health Research, Australian Catholic University, Melbourne, VIC, Australia; ^3^Department of Neurology and Immunology, Peter Duncan Neuroscience Unit, St Vincent’s Hospital, University of New South Wales, Darlinghurst, NSW, Australia; ^4^The Kirby Institute, Faculty of Medicine, University of New South Wales, Kensington, NSW, Australia; ^5^St Vincent’s Hospital, University of NSW, Darlinghurst, NSW, Australia; ^6^Royal Prince Alfred Hospital, Sydney, NSW, Australia; ^7^The Canberra Hospital, Canberra, ACT, Australia; ^8^University of Notre Dame, Sydney, NSW, Australia

**Keywords:** PASC-CI, MRI, BBB, glutamatergic excitotoxicity, diffusion tensor imaging

## Abstract

**Objective:**

To investigate the association between blood–brain barrier permeability, brain metabolites, microstructural integrity of the white matter, and cognitive impairment (CI) in post-acute sequelae of SARS-COV-2 infection (PASC).

**Methods:**

In this multimodal longitudinal MRI study 14 PASC participants with CI and 10 healthy controls were enrolled. All completed investigations at 3 months following acute infection (3 months ± 2 weeks SD), and 10 PASC participants completed at 12 months ± 2.22 SD weeks. The assessments included a standard neurological assessment, a cognitive screen using the brief CogState battery and multi-modal MRI derived metrics from Dynamic contrast enhanced (DCE) perfusion Imaging, Diffusion Tensor Imaging (DTI), and single voxel proton Magnetic Resonance Spectroscopy. These measures were compared between patients and controls and correlated with cognitive scores.

**Results:**

At baseline, and relative to controls, PASC participants had higher K-Trans and Myo-inositol, and lower levels of Glutamate/Glutamine in the frontal white matter (FWM) (*p* < 0.01) as well as in brain stem (*p* < 0.05), and higher FA and lower MD in the FWM (*p* < 0.05). In PASC participants, FA and MD decreased in the FWM at 12 months compared to baseline (*p* < 0.05). K-Trans and metabolite concentrations did not change significantly over time. Neurocognitive scores did not correlation with the increased permeability (K trans).

**Interpretation:**

PASC with CI is associated with BBB impairment, loss of WM integrity, and inflammation at 3 months which significantly but not uniformly improved at 12 months. The loss of WM integrity is possibly mediated by BBB impairment and associated glutamatergic excitotoxicity.

## Highlights

- Pathogenesis of post-acute SARS CoV-2 infection associated neurocognitive impairment (PASC CI) is unclear.
- We believe this is the first longitudinal study to show that there is blood brain barrier disruption associated with glutamatergic excitotoxicity that is likely responsible for the neurocognitive impairment.
- Our findings support further studies of these MRI techniques as potential biomarkers for PASC CI to facilitate diagnosis, appropriate timing of potential therapy and its monitoring.

## Introduction

Cognitive impairment (CI) is one of the common symptoms of post-acute SARS CoV-2 infection (PASC), otherwise known as long covid (1). The pathogenesis, however, remains unclear with possibilities ranging from immune dysregulation, autoimmunity, microthrombi, and blood–brain barrier (BBB) impairment (2, 3). To date, there is only one study which explored the role of BBB disruption using MR imaging, but the study did not have a longitudinal design (4).

Brain imaging has provided a limited contribution to understanding of CI development following COVID-19. Studies using standard MRI brain scan sequences have mostly focused on severely affected patients during acute infection showing ischemia, hemorrhage, venous sinus thromboses (2, 5, 6). There is, however, limited literature for brain imaging using more advanced techniques in PASC. Studies using structural and diffusion MRI in the post-acute phase found alterations in grey matter (GM) volume, including the hippocampus (7–10), and white matter (WM) changes (8, 10). One volumetric MRI study, which followed 401 COVID-19 patients at the onset of infection and 4 months from the acute phase, demonstrated reduced brain volume in the orbitofrontal and parahippocampal gyrus related to the primary olfactory and gustatory systems (7). A recent cross-sectional study explored BBB disruption as the cause of CI in PASC patients (4). This study used dynamic contrast enhanced (DCE) perfusion imaging and demonstrated that BBB impairment correlated with neuroinflammatory markers. Other studies have used diffusion imaging and resting state fMRI to assess brain changes in PASC with CI and showed widespread diffusivity changes along the main axis of WM tracts, and abnormal functional connectivity (FC) among resting state networks (11–16). Taken together, these findings suggest that chronic inflammation can potentially alter the BBB, brain microstructure, and neurochemicals, which may accelerate and perpetuate a chronic neuropathological change in PASC in a self-sustaining cycle, leading to cognitive impairment.

Herein we used a longitudinal design and investigated mechanistic pathways associated with the development of CI in PASC subjects. Firstly, we test using serial imaging a triple-hit hypothesis where the convergence of three deleterious factors: (1) changes in BBB, (2) altered brain neurochemicals associated with neurotoxicity, and (3) disturbed WM microstructure contribute to CI. Finally, we also explored whether these three metrices are associated with each other and the cognitive scores in the PASC participants.

## Materials and methods

Fourteen subjects with PASC who had persisting symptoms of anosmia, ageusia, fatigue, and CI who were clinically evaluated at the Neurology Department at SVH between July 2021-August-2022 were consented for the use of their clinical data for research. All 14 patients have been referred for CI post COVID and all underwent a full neurological assessment (BJB). MR imaging was conducted as part of the neurological assessment. All participants underwent routine and advanced MR imaging early in the disorder (3 months ± 2 weeks) referred as Time Point 1 (TP-1) and 10 repeated the MRI scan at 12 months (± 2 weeks) here after referred as Time Point 2 (TP-2). Seven of those participants had been enrolled into the ADAPT study, a prospective cohort of 128 SARS-CoV-2 positive patients and had received serial measurements of cognition with the Cogstate Brief Battery (1). In brief, the neurocognitive score (NCS) was derived from CogState: individual task *z*-scores were averaged into a mean *z*-score (higher equates to a better objective global performance) corrected for normative age, education and sex effects. Individuals with a prior history of drug use, significant head injury, psychiatric illness, and hepatitis C virus co-infection were excluded. Cognitively, between TP-1 and TP-2 most patients did not significantly improve suggesting that even if not strictly cognitively impaired at baseline, that most may have performed below their optimal level of cognitive functions. As such, two patients significantly improved, 5 remained stable and 3 declined TP-1 and TP-2. Out of 14 subjects only one is left handed and all the subjects including controls are right handed. Ten healthy age and sex matched controls were recruited and underwent the same neurological assessments at one time point (BJB) (Table 1). Local ethics approval was obtained (2022/ETH0022).

**Table 1:** Baseline characteristics among PASC CI cases and controls.

	Cases (*n* = 14)	Controls (*n* = 10)
*Sex*		
Male	6 (43%)	5 (50%)
Female	8 (57%)	5 (50%)
Age (years, mean, and SD)	49 (±2)	46 (±1.7)
Duration between COVID-19 diagnosis and first MRI (weeks, mean, and SD)	12 (± 1)	N/A
*Acute covid severity*		
Mild	11 (78.9%)	
Moderate	2 (14.2%)	
Severe	1 (7.1%)	
Neurological symptoms		N/A
Loss of Smell	13 (93%)	
Loss of Taste	13 (93%)	
Confusion	14 (100%)	
Tiredness/Myalgias	14 (100%)	
Neurology at TP-1		N/A
Loss of smell	13 (100%)	
Loss of taste	13 (100%)	
Tiredness	14 (100%)	
Brain fog	14 (100%)	

### Image acquisition

All imaging was performed with a 3TMR imaging scanner (Ingenia; Philips Healthcare, Best, the Netherlands) with a 24-channel head coil. DCE perfusion imaging, 32 directional diffusion imaging and single voxel MRS with short TE of the frontal cortex/ white matter (FWM) and brainstem in addition to the routine clinical imaging (T-1 volumetric imaging and T-2 FSE) were performed.

T1-weighted imaging was performed with the following parameters 3DT-1 spoiled gradient recalled acquisition in steady state (SPGR): 128 sagittal slices, 1 mm isotropic, time to repeat/time to echo (TR/ TE): shortest, field of view: 240, Matrix: 256/256.

#### DCE perfusion MRI

The DCE-MRI sequence was obtained using 3D T1-weighted spoiled gradient echo sequence in the axial plane covering the entire brain [TR and TE¼shortest (Act TR/TE 15/3.0 ms, temporal resolution 5.8, flip angle 150, matrix = 184 × 141, number of slices = 23, slice thickness = 4 mm, number of signal averages = 1, temporal resolution = 5.8/dynamic, number of dynamics = 90 and scanning time = 9.06 min. Contrast injection was commenced 6 s after the start of the dynamic MRI acquisitions, given in the form of a bolus injection of gadobutrol (Gadovist, Bayer, California, United States) at a concentration of 0.1 mmol/kg of body weight at 3 mL/s. Following the DCE-MRI scan, postcontrast-enhanced volumetric T1-weighted images were acquired as part of the routine clinical examination.

#### Magnetic resonance spectroscopy

We also obtained single-voxel MRS in the anterior FWM at the ventricular level as well as from the brainstem. These sites were chosen because previous studies had suggested that CI was more in keeping with frontal lobe dysfunction by neuropsychological testing (13) and brainstem involvement by autopsy studies at least in more severely affected patients (2, 3, 17). We did not perform whole brain spectroscopy as we were concerned at loss of resolution compared to targeted regions of interest, and the extra time required in the scanner. Single voxel 1H spectra were acquired using a short TE PRESS sequence (TE/TR 30/1,800 ms, bandwidth 2,000 Hz, 2,048 data points). A voxel size of 30/15/10 mm (AP/RL/FH) was performed in two regions: anterior frontal centrum semiovale (white matter) with adjacent cortex at the level of the frontal horns of the lateral ventricles, and brainstem. Field homogeneity and water suppression were adjusted using automated algorithms from Philips. The spectra were obtained with TE/TR¼30/1,800 ms, flip angle of 908, bandwidth¼2000 Hertz, 128 averages.

#### Diffusion tensor imaging

The DTI protocol consisted of a single-shot spin-echo-based echo-planar diffusion-weighted imaging with three averages and 36 gradient encoding directions, with b values of 0 and 1,000 s/mm2. The imaging parameters were slice thickness 5 mm, interslice gap 1.5 mm, FOV: 230 ×230, matrix 128/128, TR 3,500 and TE 96Ms.

### Image processing

#### DCE perfusion MRI

DCE studies were processed with nordicICE [nordicICE (NICE) 4.0.4; NordicNeuroLab, Bergen, Norway], a propriety software that includes brain extraction, motion correction and image registration. We assessed the DCE-derived metric K trans in multiple regions of the brain (18). The software has inbuilt features to correct the leakage correction and removal of negative slope values (values below zero), which were used to offset the blood plasma volume intercept. A two-compartment pharmacokinetic model was applied in the regions of interest (ROIs) by using the Patlak graphical approach based on linear fitting of scatter plots which was found to be the most appropriate model in a low-leakage regimen (19, 20). This Patlak graphical approach provided the BBB leakage rate and the local blood plasma volume. The slope of this fit is the BBB leakage rate (assuming a tissue density of 1 g/mL), and the intercept is the local blood plasma volume. All the patients were tested for fit of the model (chi-square goodness of model fit). Moreover, at very low-level BBB disruption, the flow is not a significant contributor to K trans (*cf.* enhancing MS plaques and tumors) (20, 21).

**Figure 1:**
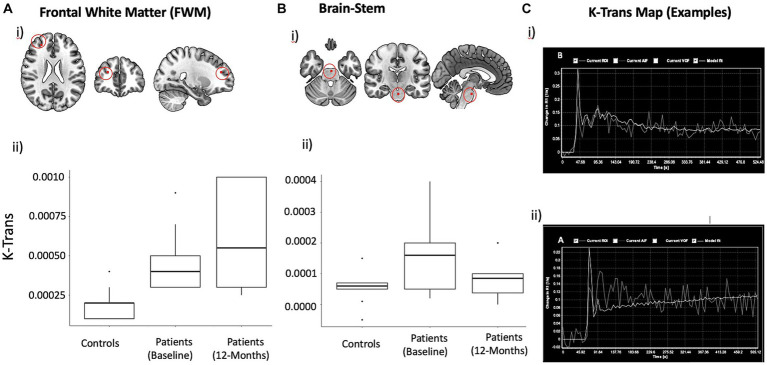
DCE derived mean K trans Maps with localization in the anatomical space and Statistical plots. K-Trans values in FWM **(A)** and brainstem **(B)**. Approximate location of voxel used for measuring K-Trans in frontal white matter is shown on a template brain (i). Box plots showing mean and inter-quartile range K-Trans values at baseline (ii) and changes over time (iii). **(C)** Representative mean k trans map demonstrating progressive change in the signal intensity (*X*-axis) over time (*Y*-axis): Normal physiological response in a healthy subject (i) and abnormal response showing increasing signal intensity (ii), indicating increased capillary permeability, are shown.

The k trans images were interrogated by placing multiple regions of interest (ROI) in the following areas of the brain [basal ganglia (caudate and lentiform nucleus), frontal cortex, frontal white matter, thalami, splenium of corpus callosum, occipital cortex and white matter, internal capsule, brainstem and cerebellar lobes) by two radiologists, one with 25 years of experience and one with 3 year of experience (JC, ML) (Figure 1). The volumes of the ROIs were 0.7 mL and whenever the area was smaller due to volume loss, the ROI was adjusted to reduce the effects of CSF. After extraction of negative values, the remaining cumulative sum of the bins was defined as the BBB leakage volume fraction. K trans values were obtained from identical regions of the brain from the opposite hemispheres and the average values were taken to compare with the normal controls (e.g., average values of both hemispheres interrogated from individual anatomical regions).

#### MRS

All spectra were taken for analysis with the LCModel (ver: LCModel: 6.3–11) using the water scaling option. Raw data were selected when the metabolites could be accepted for further statistical analysis only if the standard deviation provided by LCModel was within 20%. LCModel fits a linear combination of the full spectral pattern for each metabolite included in the base set. The absolute values of N-acetyl aspartate (NAA), creatine (Cr), glutamate/ glutamine (Glx), glutamate (Glu), glutamine (Gln), choline (Ch), and myo-inositol (MI) were calculated by the LCModel relative to free water (55 mole/L) and are reported in millimoles uncorrected for T1 and T2 relaxation.

#### DTI

DTI data were analyzed using FSL toolbox (22, 23). The pre-processing steps included denoising of DTI data based on random matrix theory, removal of Gibbs ringing artifacts, head movement, eddy current correction and bias correction. DTI parameters, including FA and MD, were calculated on a voxel-by-voxel basis by using DTIFIT tool (FSL toolbox). The Tract-Based Spatial Statistics (TBSS) analysis was employed to examine the fractional anisotropy (FA) and mean diffusivity (MD) values across multiple subjects. To achieve subject-level registration, each individual’s FA image was first aligned to the FA standard template. Subsequently, nonlinear registration was performed to further align the images to the 1 × 1 × 1 mm MNI152 space. Following the registration steps, a mean FA skeleton was generated. This skeleton represented the centers of all tracts common to the group, providing a concise representation of the white matter tracts of interest. Finally, each participant’s aligned FA maps were projected onto the mean FA skeleton. This projection step ensured that the analysis was performed on the skeletonized FA values, effectively reducing the data to the common white matter tracts. The resulting skeletonized FA maps were then subjected to voxel wise group-level analysis, examining differences in DTI parameters between groups. In addition to FA, diffusivity maps based on MD were generated using the same pre-processing and registration steps outlined above. Longitudinal TBSS analysis was performed to compare FA and MD between two timepoints.

### Statistical analysis

All statistical analyses of K-Trans and brain metabolites were performed using SAS statistical software (version 9.4; SAS Institute Inc., Cary, North Carolina, United States). Effect sizes were estimated using R scripts. Statistical analysis of FA and MD data was performed using randomize tool in FSL (22, 23).

Given the small size of the sample and nonnormality of data in the descriptive analyses, nonparametric analyses were used for group comparisons. A two-sample Wilcoxon rank sum test was used to compare K-Trans and Spectral metabolites between the patients and controls. Wilcoxon signed-rank test (for repeated measures) was used to compare K-Trans and spectral metabolites between two timepoints. The tests were considered significant at *p* < 0.05. The effect sizes for Wilcoxon tests were calculated by dividing the *Z*-scores by the square root of sample size.

For the statistical analysis of the FA and MD data obtained from TBSS analysis a general linear model was implemented for estimating between group and within group effects. The FSL function “randomize” (23, 24), was utilized for statistical analysis. Non-parametric permutation testing with 5,000 permutations was employed for statistical testing. A threshold-free cluster enhancement (CLUSTER) method was used for the correction of multiple comparisons and any differences at *p* < 0.05 (CLUSTER corrected) were considered to be significant.

### Exploratory correlation analysis

Exploratory correlation analyses were performed between K-Trans as an independent variable and NCS, spectral metabolites (Glx) and diffusion metrics (fractional anisotropy-FA) as dependent variables obtained from the brainstem and frontal white matter. Spearman’s correlation, a non-parametric method, was used to establish the strength of the relationship between the variables. First, we ran correlations between K-Trans and the other variables between the patients and controls and subsequently within patients only, taking into cognizance that the patient data alone has a smaller sample size and may not have the strength to show the effects conclusively.

## Results

### Changes in blood brain permeability (K-trans)

Figure 1 summarizes the mean K-trans measurements in the PASC CI and control participants in the frontal white matter and brainstem. At baseline, PASC CI participants exhibited significantly higher K-Trans values compared to controls in the FWM (effect size (*r*) = 0.72, *p* < 0.01) and brainstem (*r* = 0.44, *p* = 0.03). Within PASC CI participants, there was no significant change in K-Trans values over time (Figure 1).

### Changes in brain metabolites

**Figure 2:**
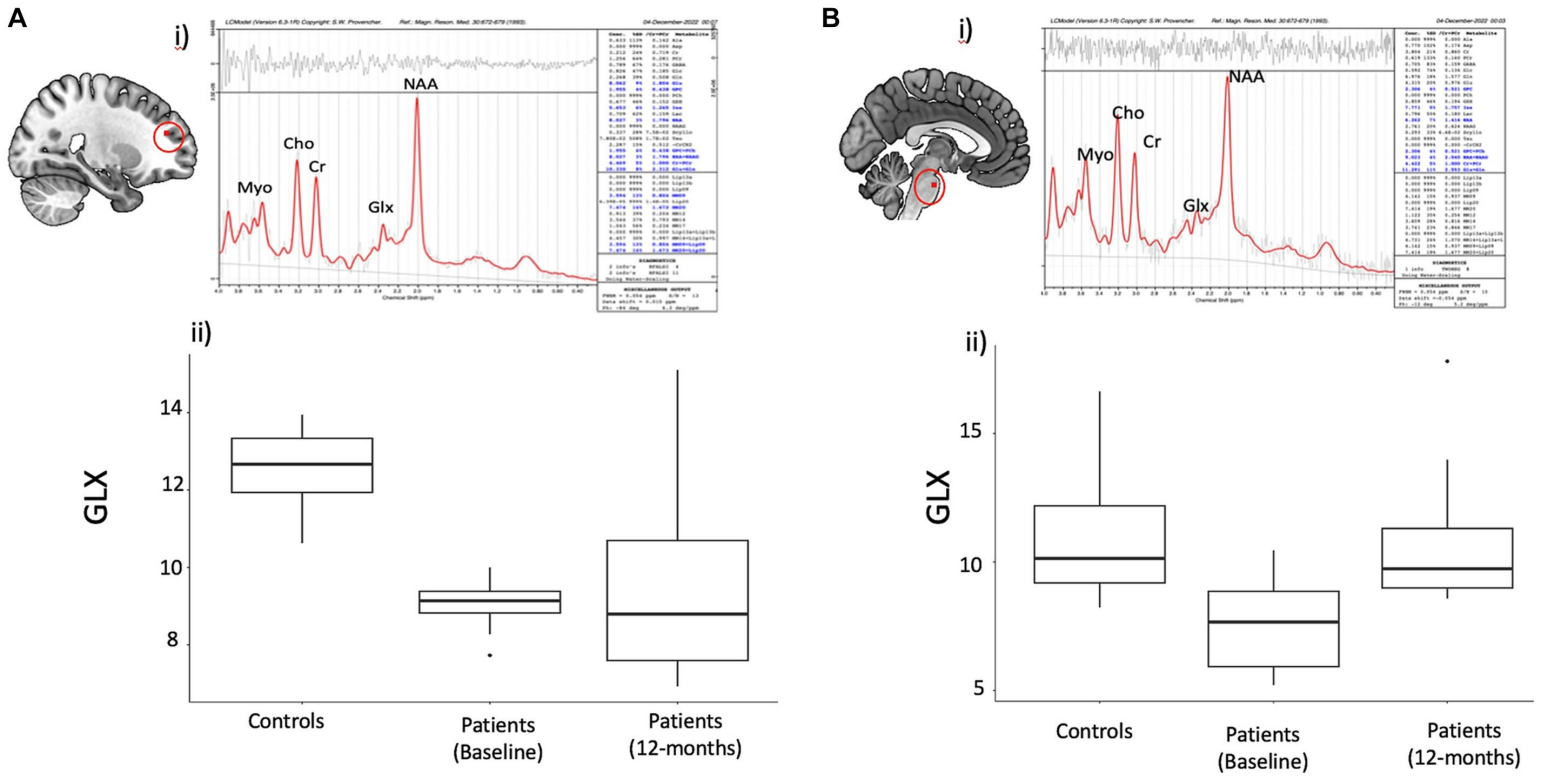
Spectral data in the frontal region and Brain stem with statistical observations in the Covid subject’s vs. controls. Brain metabolite changes in patients compared to controls. **(A)** (i) Representative spectra of single voxel short TE MRS in the frontal region using LC model. (ii) Boxplot showing difference in frontal white matter (FWM) GLX values between patients and controls (iii) Boxplot showing GLX change over time within patients **(B)** (i) Representative spectra of single voxel short TE MRS in the brainstem region using LC model. (ii) Boxplot showing difference in brainstem GLX values between patients and controls (iii) Boxplot showing change over time in patients in the brainstem GLX in patients.

Figure 2 summarizes the metabolites measurements in the PASC CI and control participants in the FWM and brainstem. At baseline, PASC CI participants exhibited significantly lower GLX values compared to controls in the FWM (effect size (*r*) = 0.83, *p* < 0.01) and brainstem (*r* = 0.67, *p* < 0.01). Among PASC CI patients, there was no significant change in frontal GLX values over time, however, brainstem Glx values increased (*r* = 0.75, *p* = 0.01) over time.

Other metabolites showed inconsistent changes across FWM and brainstem. PASC CI participants exhibited significantly higher Myo values compared to controls in the FWM (effect size (*r*) = 0.60, *p* = 0.004), but not in the brainstem (*r* = 0.07, *p* = 0.73). NAA values decreased in the brainstem (*r* = 0.49, =0.02), as well as in the frontal white matter but only at trend level (*r* = 0.39, *p* = 0.06).

### Changes in white matter microstructure

**Figure 3:**
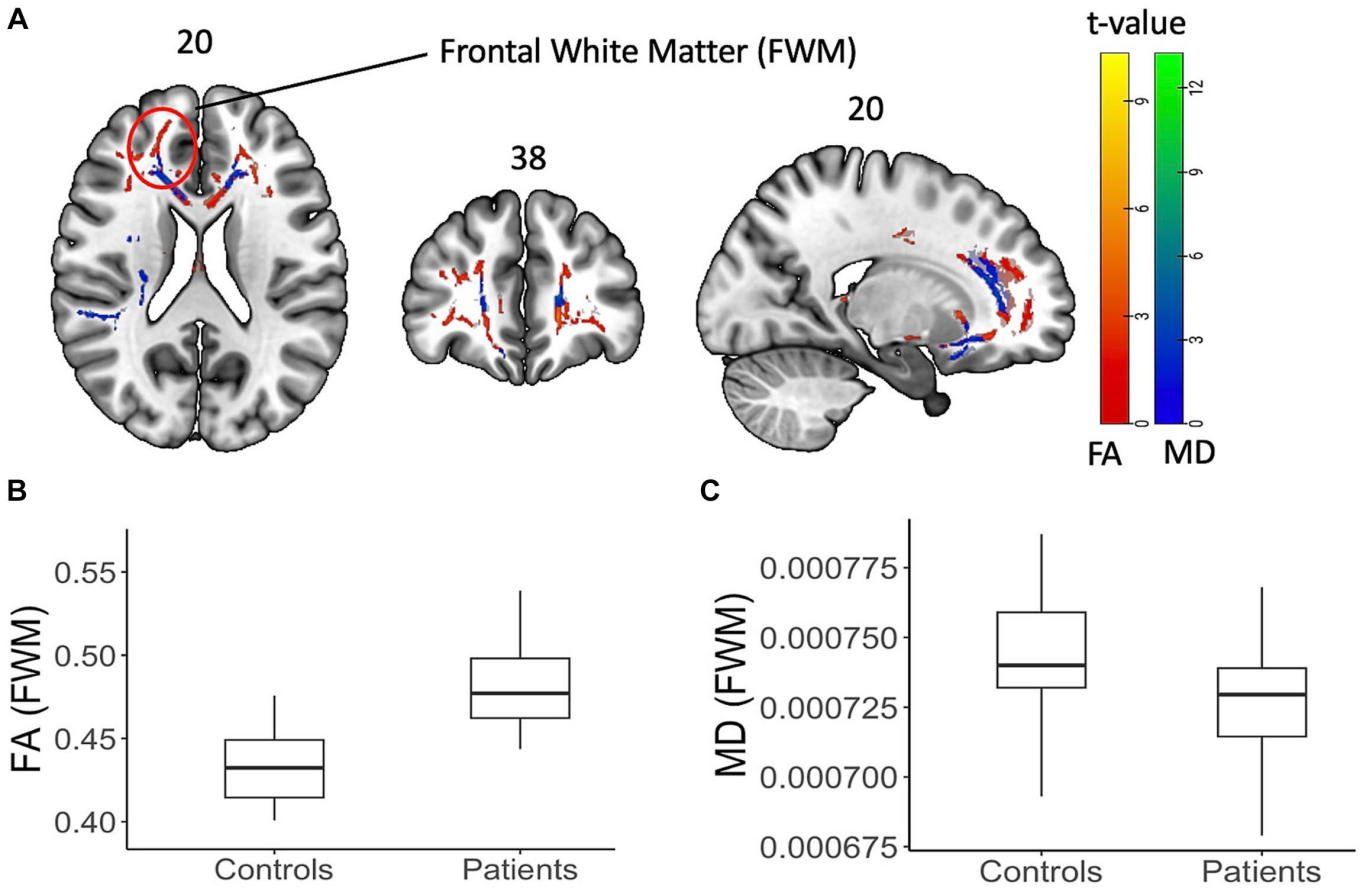
Diffusion tensor data with FA and MD on a shaded surface display and statistical maps between subjects and controls. DTI-based changes in fractional anisotropy (FA) and mean diffusivity (MD) values in patients compared to controls. **(A)** TBSS analysis showed significant (*p* < 0.05, cluster corrected) increase (red) in FA values and decrease (blue) in MD values. The color bar represents corresponding *t*-values. **(B,C)** Box plot of FA and MD values from an example FWM area (as highlighted on the brain image) showing the difference.

Figure 3 summarizes the findings from the TBSS analysis of DTI data at baseline. At baseline, PASC CI participants exhibited significantly increased FA (*p* < 0.05, cluster corrected) and decreased MD (*p* < 0.05, cluster corrected) in widespread regions, such as FWM, corpus callosum, cerebral peduncles, and sagittal striatal white matter compared to controls. The white matter regions showing differences and corresponding effect size (*t*-values) are provided in Table 2.

**Table 2:** Average *t*-values within the white matter regions showing significant increase in FA from TBSS analysis (*p* < 0.05, cluster corrected).

Atlas label	*t*-value
Sagittal sriatum L	3.95
Anterior corona radiata L	3.51
Superior cerebellar peduncle L	3.22
Superior fronto-occipital fasciculus R	3.06
Genu of corpus callosum	2.95
Sagittal stratum R	2.94
Cerebral peduncle L	2.59
Inferior fronto-occipital fasciculus R	2.59
Superior longitudinal fasciculus L	2.48
Fornix (CRES)/Stria terminalis L	2.37
Inferior cerebellar peduncle R	2.32
Cerebral peduncle R	1.94
Superior longitudinal fasciculus R	1.94
Medial lemniscus R	1.92
Superior corona radiata R	1.70

These regions were based on JHU ICBM DTI atlas.

**Figure 4:**
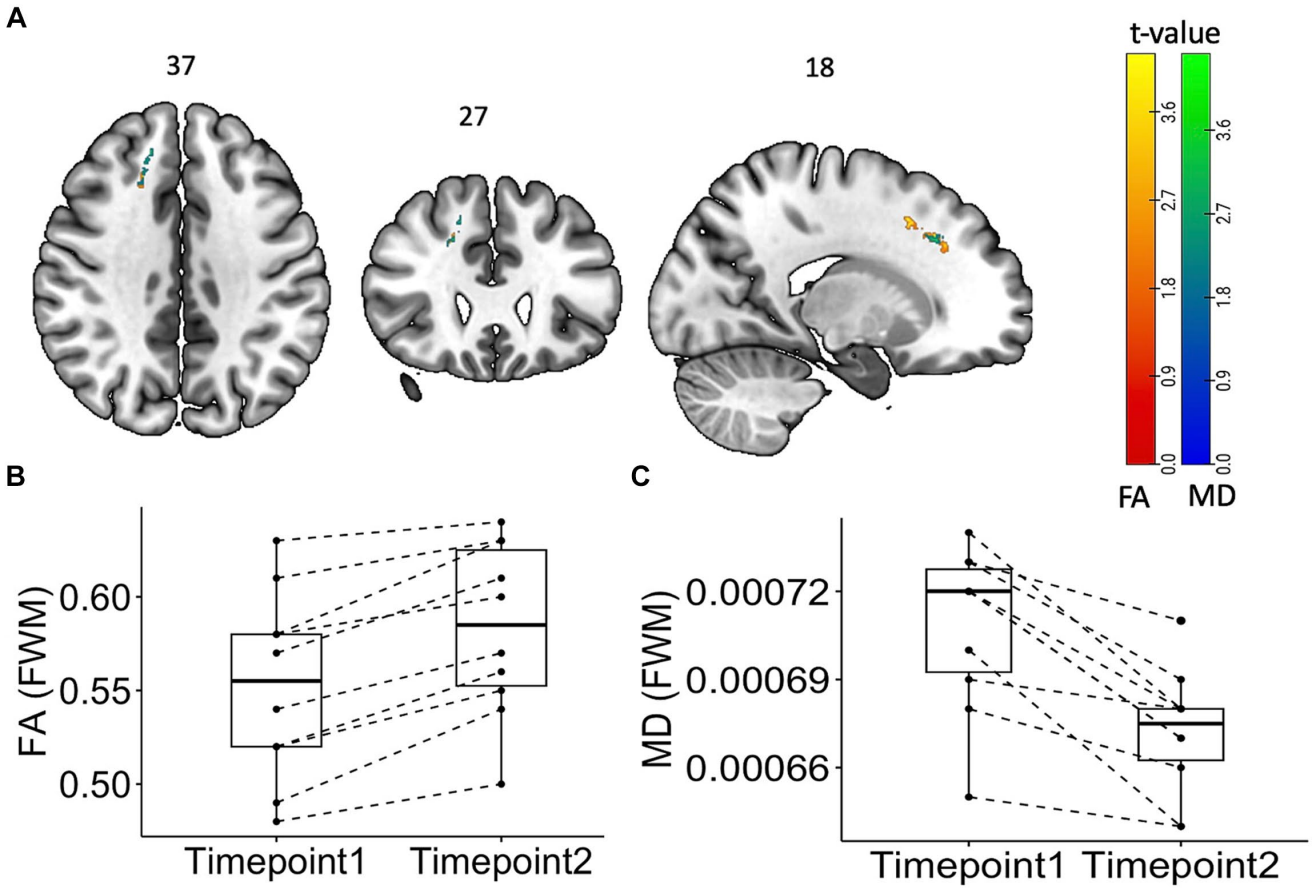
Tract Based Spatial Statistics (TBSS) Maps showing time change “within” the subject group with statistical representation. Longitudinal TBSS analysis showing changes in fractional anisotropy (FA) and mean diffusivity (MD) values over time in patients. **(A)** TBSS analysis showed significant (*p* < 0.05, cluster corrected) increase (red) in FA values and decrease (blue) in MD values. The color bar represents corresponding *t*-values. **(B)** Box plot of FA and MD values from the frontal white matter area showing the difference.

Figure 4 summarizes the findings from the longitudinal TBSS analysis among PASC CI participants. There was significant increase in the FA and reduced mean diffusivity (*p* < 0.05, cluster corrected), in the superior longitudinal fasciculus, a long white matter bundle associated with executive function.

### Correlation with neurocognitive scores

**Figure 5:**
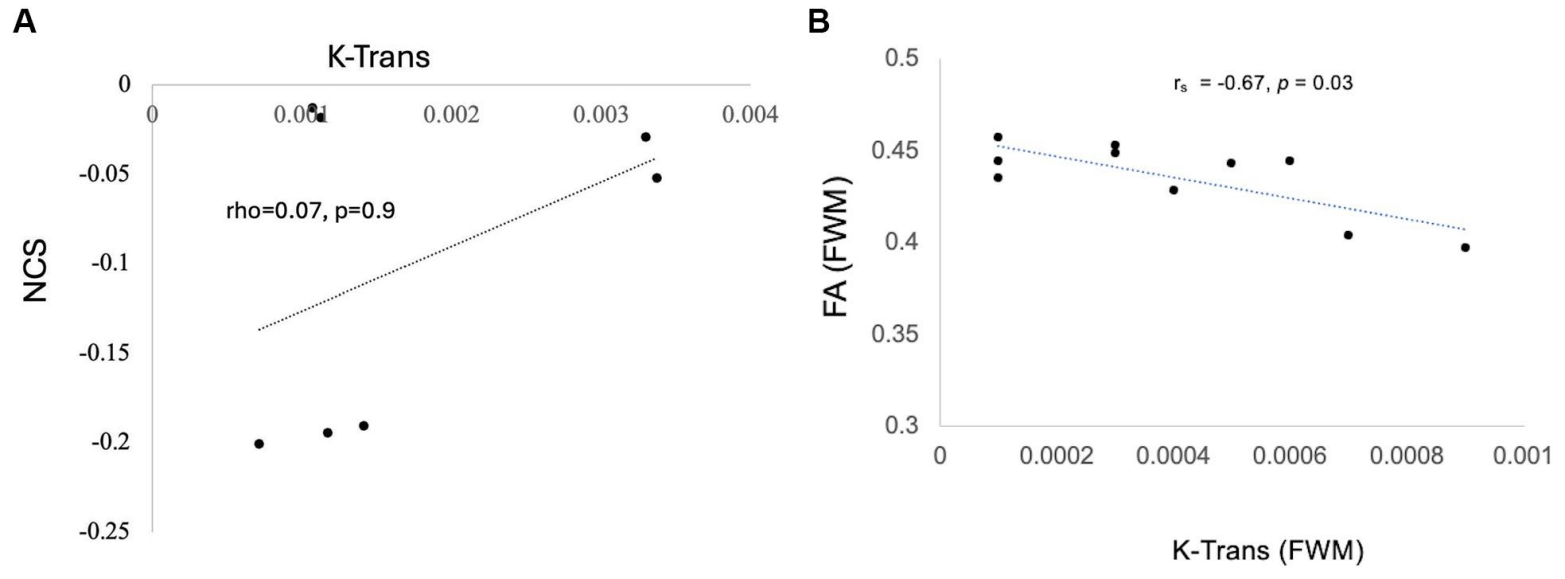
Correlation of K trans with NCS **(A)** Trend-level correlation between brainstem K-Trans and NCS and **(B)** statistically significant correlation of k trans and FA in the FWM.

Exploratory correlations were performed between K-Trans, brain metabolites, and cognitive scores. In the PASC CI participants (7 patients), there was a no correlation between brainstem K-Trans and neurocognitive scores (rho = 0.07, *p* = 0.9). However, there was a negative correlation between FWM K-Trans and FA (*r*s = −0.67, *p* = 0.03) and trend-level correlation between brainstem K-Trans and Glx (*r*s = 0.49, *p*= 0.07). No other correlations were significant (Figure 5).

## Discussion

We have undertaken a comprehensive proof-of-concept prospective case–control study to investigate parallel changes in blood brain barrier permeability, neurochemicals, and white matter microstructure changes in PASC CI patients. We found significant differences in all three measures in PASC CI patients compared to controls: K-Trans was increased and Glx showed reduction in both the FWM and brainstem. Furthermore, we also found increased FA and decreased MD in FWM. However there is no statistically significant correlation was found between K trans and cognitive performance. Over 12-months, Glx values were normalized in the PASC CI patients, whereas the K-Trans did not significantly change. Interestingly, over 12-months, FA values further increased, and MD decreased in in the superior longitudinal fasciculus in the region of the FWM. Taken together, these findings support a pathogenic mechanism for PASC CI in which excitotoxicity is driving neuroinflammation.

Our central finding is BBB impairment as assessed by the DCE-derived metric K-trans. To the best of our knowledge, there is only one other study that has used K-trans to measure the BBB integrity in PASC (4). It also showed diffuse BBB impairment at four months post COVID-19 infection but change over the subsequent months was not examined (4). Subtle BBB disruption cannot be identified on routine MR studies with gadolinium administration. K-trans has been used in other neurological diseases characterized by BBB impairment (24) and is accepted as a marker of subtle BBB impairment at the capillary level before there is gadolinium enhancement on standard MRI sequences (25).

Both cortical grey and white matter injury have been a consistent feature of PASC (11, 26, 27), even in those with mild acute COVID-19. However, these studies have shown variable outcomes in DWI metrics with some showing increased FA and decreased MD (16) and others the opposite (7, 14). We used skeletonized FA and MD and found that FA increased, and MD decreased at baseline. There was further increase in FA at 12 months especially in the superior longitudinal fasciculus, a white matter bundle that has been shown to be closely linked to poorer working memory (28). Skeletonized MD can be used to detect small vessel disease that have been implicated in the pathogenesis of long COVID (3, 10). Interestingly, although several of these studies have shown increase in MD, we showed decreased MD and increased FA at four months. This paradoxical increase in the FA and MD has been hypothesized to be associated with axonal degeneration (29), microgliosis and inflammation (30), and was also observed in the Lu et al. study (16). This study also showed an increase in MD in a sub-group of patients who had COVID-19 encephalitis. Our study has shown similar observations, with increased FA and decreased MD across several white matter tracts in the PASC CI participants as compared to the controls at four months. The further increase in FA in the superior longitudinal fasciculus is an interesting finding, as we would have expected any white matter microstructural injury to have normalized by 12 months. Notably, this change was not in the white matter area showing significant increase compared to controls at baseline. Thus, whether this longitudinal change reflects further inflammatory changes or improved integrity (31) of the white matter over time remains unclear. This is an important consideration for future research, as the superior longitudinal fasciculus has long bidirectional projections among the prefrontal, temporal, occipital, and parietal cortices and strongly associates with perceptual organization and working memory (28).

Our results can point to pathogenesis. The spectral metabolite changes with reduced Glx and NAA point to excitotoxic neuronal injury which may be secondary to BBB impairment given the relationship to K-trans. Excitotoxicity is implicated in a number of neurocognitive disorders (25, 32) and reflected in MRS metabolites as a reduction in Glx: intracellular Glx levels are increased leading to cell injury and consequent Glx depletion within the cell. To our knowledge, there is only one other study that has examined MRS in PASC CI. This study reported increased Glx compounds. However, it did not specify the precise site of interrogation used to derive the metabolites, was cross-sectional and only expressed each individual metabolite level as a ratio to creatine. Nonetheless, it is known that excess intracellular Glx compounds lead to cell injury and consequent intracellular Glx depletion (33, 34).

There are several limitations in our study. One notable limitation of our study is the small sample size, which may reduce the generalizability of the findings to a broader population. The absence of control data at 12-months is another significant limitation, as it precludes us from making direct comparisons between patient changes and potential normative brain alterations. Additionally, the small number of patients with neuropsychological assessment and the narrow mild range of impairment likely precluded correlation of MR parameters and cognitive impairment. Despite these limitations, our study provides valuable insights into the intra-individual changes over time; however, future research with larger sample sizes and longitudinal control groups is warranted.

In conclusion, our findings have relevance to both pathogenesis and clinical evaluation. MRI techniques addressing BBB disruption, white matter integrity and inflammation may offer further insights into the steps in PASC CI pathogenesis. Further, these techniques may serve as a biomarker for the disorder, assist in the appropriate timing of a therapy, and in assessing therapeutic response.

## Data availability statement

The raw data supporting the conclusions of this article will be made available by the authors, without undue reservation.

## Ethics statement

The studies involving humans were approved by the Ethics Committee of St Vincent’s Hospital (2022/ETH0022). The studies were conducted in accordance with the local legislation and institutional requirements. The participants provided their written informed consent to participate in this study.

## Author contributions

JC: Conceptualization, Data curation, Formal analysis, Investigation, Methodology, Project administration, Software, Writing – original draft, Writing – review & editing. GP: Formal analysis, Investigation, Writing – review & editing. LC: Formal analysis, Project administration, Writing – review & editing. GD: Supervision, Writing – original draft. AK: Investigation, Writing – review & editing. GM: Writing – review & editing, Investigation. DD: Writing – review & editing. AB: Investigation, Validation, Writing – review & editing. DJ: Visualization, Writing – review & editing. XZ: Writing – review & editing, Data curation, Methodology. ML: Data curation, Writing – review & editing. NJ: Formal analysis, Writing – review & editing. BB: Conceptualization, Investigation, Supervision, Writing – original draft, Writing – review & editing.
